# Evaluation of Candidates for Systemic Analgesia and General Anesthesia in the Emerging Model Cephalopod, *Euprymna berryi*

**DOI:** 10.3390/biology12020201

**Published:** 2023-01-28

**Authors:** Skyler Deutsch, Rachel Parsons, Jonathan Shia, Sarah Detmering, Christopher Seng, Alyssa Ng, Jacqueline Uribe, Megan Manahan, Amanda Friedman, Gabrielle Winters-Bostwick, Robyn J. Crook

**Affiliations:** 1 Department of Biology, San Francisco State University, San Francisco, CA 94132, USA; 2 Department of Biology, Northeastern University, Boston, MA 02445, USA

**Keywords:** analgesia, anesthesia, cephalopod, *Euprymna*, mollusc, squid, welfare

## Abstract

**Simple Summary:**

Analgesia and anesthesia in aquatic animals can be challenging, and are exceptionally difficult to implement and evaluate in invertebrate species. In this study we test multiple analgesic candidates in the small Hummingbird Bobtail Squid, to identify drugs that may be effective at enhancing welfare in cephalopods. Cephalopods are growing in popularity as comparative neuroscience models, and there is a pressing need to refine procedures to permit their ethical use. In addition to identifying analgesic candidates for cephalopods for the first time, we also validate a general anesthesia protocol for *E. berryi* that has been tested in other cephalopods.

**Abstract:**

Cephalopods’ remarkable behavior and complex neurobiology make them valuable comparative model organisms, but studies aimed at enhancing welfare of captive cephalopods remain uncommon. Increasing regulation of cephalopods in research laboratories has resulted in growing interest in welfare-oriented refinements, including analgesia and anesthesia. Although general and local anesthesia in cephalopods have received limited prior study, there have been no studies of systemic analgesics in cephalopods to date. Here we show that analgesics from several different drug classes may be effective in *E. berryi*. Buprenorphine, ketorolac and dexmedetomidine, at doses similar to those used in fish, showed promising effects on baseline nociceptive thresholds, excitability of peripheral sensory nerves, and on behavioral responses to transient noxious stimulation. We found no evidence of positive effects of acetaminophen or ketamine administered at doses that are effective in vertebrates. Bioinformatic analyses suggested conserved candidate receptors for dexmedetomidine and ketorolac, but not buprenorphine. We also show that rapid general immersion anesthesia using a mix of MgCl_2_ and ethanol was successful in *E. berryi* at multiple age classes, similar to findings in other cephalopods. These data indicate that systemic analgesia and general anesthesia in *Euprymna berryi* are achievable welfare enhancing interventions, but further study and refinement is warranted.

## 1. Introduction

Welfare refinements for cephalopod molluscs are necessary to improve ethical treatment of invertebrate animals in research. Increasing interest in cephalopods as comparative models of complex brains and behaviors has resulted in explosive growth in cephalopods, both in terms of raw numbers and in the variety of different species, present in research labs worldwide [1]. Regulations governing the use of cephalopods in research have also expanded considerably in the past 20 years, such that cephalopods are now regulated at or near equivalent levels to those of vertebrate animals in the European Union (under Directive 2010/63/EU; [2]), the United Kingdom (under the newly-created Animal Sentience Bill; [3]), Canada (Canadian Council on Animal Care; (1996) [4], Australia (National Health and Medical Research Council, 2013); and New Zealand (NZ Animal Welfare Act, 1999), but notably, at the time of writing no such laws govern the use of cephalopods in research laboratories in the USA, where the definition of an “animal” under the Public Health Service Policy on Humane Care and Use of Laboratory Animals is explicitly restricted to vertebrate animals only (but see [5,6]). 

Where cephalopods are included in regulatory frameworks governing animals in research laboratories, their inclusion is based primarily on the precautionary principle [7] In almost all cases (except for the very recent passage of the UK Animal Sentience Bill), legislation including cephalopods as regulated species was enacted prior to scientific evidence for nociception, pain or suffering in cephalopods. However, in recent years evidence has mounted that cephalopods have neural circuits for nociception [8,9,10,11,12] and sufficient neural complexity of these circuits to support affective pain experience [13] Given the growth of cephalopods as emerging neurobiological and genetic models, the increasing regulation of invertebrate species in research, and the accumulating evidence for the existence of pain-like experience in cephalopods, the need for improved analgesia and anesthesia protocols is of pressing importance [14].

Sepiolid squid are popular research and display organisms due to their small size, tolerance for high-density housing and ease of captive breeding. In the past ten years the *Euprymna* genus has emerged as a genetic model candidate, and there is intensive study currently of its genome [15], nervous system [10,11,16], and behavior [17,18,19] Of the two primary species of *Euprymna* common in research labs, *Euprymna scolopes* has a long history of use in studies of symbiosis and host-microbe interactions, as a result of its unique co-evolution with a bioluminescent microbe which is cultured by the squid in a specialised light organ [20,21,22,23,24]. *E. scolopes* is challenging to rear in captivity, with rearing success rates varying from 50% down to almost zero [25] In contrast, the slightly larger species *Euprymna berryi* is considerably easier to rear, permitting rearing successes up to 90% of hatchlings raised until adulthood [26] and R. Crook, unpubl.). Sepiolid squid are distinct in physiology and behavior from other squid genera and from cuttlefish, suggesting that their responses to anesthesia and analgesia may differ in important ways too. While several previous studies have established protocols for effective and safe general anesthesia in cuttlefish, octopus and loliginid squid [27,28,29], no studies to date have tested these procedures in sepiolid squid. Additionally, there are no published reports of analgesia in cephalopods beyond demonstrations of local, injectable anesthetics [27,30], which, while effective at blocking all sensation in the infiltrated region for up to four hours, do not provide whole-body analgesia or permit normal processing of non-painful stimuli.

Thus in this study we aimed to identify methods for general anesthesia in this emerging model genus and for the first time, we also test potential pain-relieving effects of established veterinary analgesics from several distinct drug classes.

## 2. Materials and Methods

### 2.1. Animals

Hummingbird bobtail squid (*Euprymna berryi* Sasaki 1929, Figure 1) were captive-bred second and third-generation hatchlings from brood stock purchased from the Marine Biological Laboratory Center for Cephalopod Culture (Massachusetts, USA), in September 2021. Squid (*n* = 167) were fed ad libitum on live mysid shrimp (*Mysidopsis bahia*) until about 5 weeks post hatching, and thereafter on live grass shrimp (*Paeneus* spp.) at a rate of one 1–2 shrimp per squid per day. Squid were maintained in a recirculating seawater system (1600 L) held at 23.5–25.5 °C and filtered via physical, chemical and biological filtration. Squid were reared in floating tub enclosures (30 cm diameter and 8 cm deep) at a density of 50 hatchlings/tub, which was reduced as the squid grew to 1–2 squid per tub as adults. Tubs and main tanks contained sand beds to permit normal burying behavior and various enrichments such as plastic plants, coral rubble, flat rocks and PVC tubes, creating a naturalistic environment allowing the exercise of the normal behavioral range of this species. Squid reared in these conditions attain sexual maturity at around 4 months post hatchling and a maximum size of ~4 cm mantle length by around 6 months of age, which was the typical age of senescence and death for both sexes. Squid were monitored daily for general health, and any squid showing signs of compromised health (skin damage, evidence of impaired swimming, inability to capture live prey or other sign of poor health), were monitored and euthanized if welfare was judged to be compromised. Experiments were conducted between November 2021 and December 2022.

Ethical Note: Squid are invertebrates and therefore are excluded from regulatory oversight in the USA; thus, no IACUC protocol was required for this study. However, we adhered to Directive 2010/63/EU and ARRIVE guidelines for characterizing standards of care, humane endpoints, and experimental procedures. Efforts to minimize pain, suffering, distress and lasting harm (PSDLH, see [31]) as a result of experimental procedures were made at the design stage of the study. Table 1 shows a breakdown of squid used in different stages and the degree of severity of each procedure. In general anesthesia experiments, we used doses and procedures we had tested previously on other cephalopod taxa with minimal adverse effects [27,28]. In tests of analgesic candidates we employed a conservative approach of screening drugs by assessing nociceptive thresholds in normal (uninjured) squid, using von Frey filaments to test response thresholds, which results in very brief and always escapable noxious sensory input. Squid were sedated for all handling and injection procedures and were not reused in behavioral studies for at least two weeks. We then used the data from the von Frey threshold tests to select a subset of drugs to test in electrophysiology experiments, limiting the number of squid we euthanized in that phase of the study. Finally, we selected drug candidates based on electrophysiology and behavior to test with lasting noxious stimulation in a small number of squid, to limit the overall total of potential pain and distress involved in the study. We used moderately stringent statistical procedures (see the Section 2.9 below “Data Analysis and Statistical Procedures”) to capture large and moderate effect sizes, tolerating a slightly inflated beta error rate in favor of requiring larger sample sizes.

### 2.2. Analgesic Drugs and Dosages

We identified candidate analgesics from published reports of efficacy in fish and other vertebrates [32,33,34,35]. We selected drugs from several different drug classes and based dosing on published studies in fish if available, and then in rodents. We chose an initial drug dose toward the upper end of doses reported to be effective in other species, and in cases where we observed possible effects on squid behavior, we also tested 10× the initial dose chosen. Doses are reported as ranges in cohorts of squid where size was variable, or as a single value where cohorts for that drug were similar sizes.

Drugs chosen were as follows: Buprenorphine (“Buprenex”, an opioid), Ketorolac (an NSAID), Ketamine (a dissociative anaesthetic with analgesic properties), Dexmedetomidine (“Dexdomitor”), an alpha2 adrenoreceptor agonist) and acetominophen (a COX-2 inhibitor). Drugs were obtained either through a retail pharmacy (Rite-Aid, San Francisco, CA) (acetominophen, ketorolac) or from Covetrus Veterinary Supply (Phoenix, AZ, USA) (Buprenex, Dexdomitor, Ketamine). All drugs except for acetominophen were delivered via intramuscular injection into the base of the arm crown on the dorsal body surface. Acetominophen was dosed orally by injecting the drug solution into a live shrimp, which was then fed immediately to the squid. All drugs were diluted in filtered, sterile artificial seawater. Control injections were vehicle (ASW) only.

### 2.3. Intramuscular Injections of Analgesic Drugs

Squid were sedated for handling and injection using a combination of 1% EtOH in SW combined with a 1:3 mixture of SW and isotonic (330 mM) MgCl_2_. After 1–2 min of immersion, squid were removed from the bath and 20 µL of sterile solution was injected into the base of arm crown, distal to the eyes and head, on the mid dorsal surface. This region is highly vascular and test solutions dyed with Fast Green dye showed circulation within 5–10 min. Injections were either of control (fASW) or drug solution (see Table 2 for drugs and dosages). Immediately after injections squid were placed individually in holding tubs for recovery, which usually occurred within five minutes. Placement of squid was by an experimenter not involved with the testing, ensuring that all testing was done blind.

### 2.4. Behavioral Assays of Analgesia Effects

At one and three hours after injections, squid were collected from holding tubs and placed into glass evaporation dishes (125 mm diameter) for tests of behavioral response thresholds. Glass testing chambers were placed on a white Styrofoam pad and surrounded by a white circular blind, limiting visual stimulation. All behavioral assays were videotaped from directly overhead with a Sony HDRCX405 Handi-Cam (Sony, New York, NY, USA). After two minutes of acclimation, an ascending series of von Frey filaments was applied by hand to the dorsal mantle at the point where the anterior fin margin meets the body. We used eight von Frey filaments in ascending series (0.02, 0.04, 0.16, 0.6, 1, 4, 10, and 26 g). Filaments were applied only when the squid was sitting on the bottom of the glass chamber, not swimming, so intervals between filament applications were variable and based on squid behavior. Trials varied in length from <5 min to 60 min, which was set as the maximum duration. Squid were returned to their enclosures if no threshold has been established by 60 min.

Behaviors were classified as either mechanosensory responses (chromatophore change, avoidance swimming, arm movement) or nociceptive responses (jetting, inking, stimulus-directed arm grooming), and the filament at which each behavior occurred was noted. Once nociceptive threshold was reached no further stimulation was applied.

### 2.5. Electrophysiological Measures of Analgesia

Squid that had not been used in previous behavioral studies were used for electrophysiological recordings of pallial nerve excitability. Squid were killed by terminal anesthesia via immersion in isotonic MgCl₂ for 15 min, followed by rapid decapitation and decerebration. Dissections were carried out in a mixture of 1:1 fASW and isotonic MgCl_2_. Pallial nerves were exposed via a ventral midline incision in the mantle, removal of viscera and then careful removal of overlying connective tissue around the stellate ganglion and pallial nerve. The mantle was bisected down the dorsal midline to create left and right preparations, which were treated either with drug or control solutions. Preparations were pinned tightly into Sylgard-lined dishes and treatment solutions washed on to each preparation by an experimenter not involved in the recording, thus all electrophysiological recordings were conducted blind. Preparations were left to incubate in the test solutions for 60 min, then recordings were carried out in fresh washes of the same solutions.

To record peripheral nerve excitability, pallial nerves were drawn into a suction electrode connected to an A-M Systems (Sequim, WA, USA) model 1700 extracellular amplifier. Traces were digitized and sampled at 20 KHz with a Powerlab 4/35 (AD Instruments, Sydney, Australia). Prior to any stimulation of the tissue, two minutes of spontaneous activity was recorded, then the mantle tissue was stimulated with three touches of a light filament (0.16 g) to activate mechanoreceptors, immediately followed by three replicate touches of a heavy filament (4.0 g) to activate nociceptors. This recording block was repeated after five minutes.

### 2.6. Responses to Painful Fin Pinch

We tested three drug candidates (ketorolac at 3 mg/kg, buprenorphine at 0.15 mg/kg and dexmedetomidine at 5 µg/kg) in this phase of the study, based on combined evidence from baseline tests of nociceptive thresholds and electrophysiological recordings of pallial nerve excitability. Injection procedures were as described above. One hour after injection, squid were placed in individual glass evaporation dishes and allowed to acclimate for two minutes. A single firm pinch was delivered using grooved forceps to one fin margin, then behavior was recorded for 10 min after pinch. We counted instances of inking, wound-directed grooming and ventilation rates, immediately after pinch and again at 1, 5, and 10 min post-pinch.

### 2.7. Sequence Alignment Methods

Multiple sequence alignments were created using default parameters for Muscle [36] in Mega version 11 [37] Publicly available sequences were identified using BLASTp [38] searches on NCBI or the tBLASTn function on Sequenceserver [39] hosting *Euprymna scolopes* sequence data from [40] Accession numbers for Genbank sequences used for alignments: Hs_ α2A: AAF91441.1, Dr_ α2A: NP 997520.3, Ob_ α2A: XP_014767930.1, Hs1_NMDA1: XP 005266128.1, Dr1_ NMDA1: XP_005171833.1, Ob1_NMDA1: XP_014790759.1, Hs2a_NMDAR2A: 6IRF_B, Hs2b_NMDAR2B: 7EU8_B, Dr2_NMDAR2: XP_021329529.1, Ob2_NMDAR2: XP_014786064.1, Hs_PGS: AAA03630.1, Hs_COX2: AAA58433.1, Dr_PGS: NP_705942.1, Ob_COX_PGS: XP_014768109.1, Sk_OctR: XP 002734062.1, Ob_OctR: XP 014778476.1, Sk_TyrR: XP 002733591.1, Ob_TyrR: XP_014791053.1, 1. Accession numbers for *E. scolopes* Sequenceserver sequences used for alignments:

Es_TyrR: Lachesis_group41__39_contigs__length_54644242,

Es_COX_PGS: Lachesis_group29__46_contigs__length_89259793,

Es2_NMDA2: Lachesis_group19__43_contigs__length_108131866;

Es_ α2A: Lachesis_group20__56_contigs__length_105542877,

Es1_NMDA1: Lachesis_group10__55_contigs__length_138218214,

Es_OctR: Lachesis_group20__56_contigs__length_105542877.

*Euprymna berryi* sequences were identified using local tBLASTn from Crook Lab Illumina RNA-sequencing on the *E. berryi* stellate ganglia. Selected sequences were submitted to NCBI Genbank and provided the following accession numbers: Eb1_NMDA1: OQ106914, Eb2_NMDA2: OQ106915, Eb_COX_PGS: OQ106910, Eb_OctR: OQ106912, Eb_TyrR: OQ10691.

Sequence alignment images were created using GeneDoc software and residues with functional significance were identified from published literature [41,42,43] and highlighted manually in Microsoft Word 16.7 for Mac.

### 2.8. General Anesthesia Trials

We used drug combinations validated previously as effective in cuttlefish and octopus [27,28], and which were recently shown to be viable in loliginid squid [29] We focused on a mix of 1.0% EtoH (*v*/*v*) combined with a 1:3 mixture of 330 mM MgCl_2_:ASW, and measured induction and reversal times in hatchlings, sub-adult and senescent squid.

Squid were removed from their home tank enclosures and placed individually in a glass evaporation dish (125 mm diameter), which was filled with home tank water. All trials were videotaped from directly overhead. After one minute of continuous recording, 25% of the seawater was siphoned out and replaced by isotonic MgCl_2_ solution, making a 1:3 ratio mix with seawater. Immediately, ethanol was also added at a concentration of 1.0% of the total bath volume. Squid were monitored for signs of increasing anesthesia, including loss of chromatophore tone, cessation of swimming movements and loss of righting reflex. As soon as the squid was determined to be anesthetized the anesthetic bath was replaced completely with fresh ASW and reversal monitored until the squid showed normal behavior. Survival of all squid was recorded at 24 h post testing. Adult and senescent squid were tested individually. Tests of hatchling squid were done in batches of eight squid per trial.

### 2.9. Data Analysis and Statistical Procedures

Baseline detection and nociceptive thresholds were analyzed from recorded video footage. Videos were analyzed separately by trained analyzers for concurrence and rescored by the first author (S.B.D.) and senior author (R.J.C.) if observers’ measurements differed. We used one-way ANOVA followed by unpaired (independent) *t*-tests to compare thresholds among control and treatment squid in each experimental cohort. To assess whether drugs showed evidence of sedative effects (which could result in diminished arousal and responsiveness, and thus could be misinterpreted for analgesia), we compared the total trial length among all groups. Because filaments were applied only when the squid was quiescent and sitting on the bottom of the dish (not swimming or moving around), inter-stimulus interval (and thus total trial length) was primarily determined by squid activity level. We hypothesized that longer trials indicated greater activity levels, and shorter trials suggest reduced activity or arousal levels. Shorter trial length may thus indicate a sedative effect of the drug being tested. We compared total trial length with a one-way ANOVA.

For electrophysiological data, traces were median filtered and spikes above the noise threshold were counted automatically using the “Spike Histogram” module 2.6.3 of LabChart v8.0 (AD Instruments, Sydney, Australia). For spontaneous activity, we measured total spikes in one minute of recording. For evoked responses, we counted spikes from one second of maximum firing during filament application. Within each recording block, counts from the three replicate touches were averaged, then a grand mean was computed from the average counts from the two test blocks. Counts of spontaneous activity were compared with independent samples *t*-tests (each treatment group compared to controls), and counts of evoked firing were compared with paired *t*-tests, where one side of the mantle was the control and one side was treated with a given drug.

Pain-like behavior in response to strong noxious stimuli (fin pinch) was video recorded, and ventilation rates, along with counts of inking and directed grooming, were measured immediately after pinch, and again at one, five, and 10 min post-pinch. Frequency of behaviors from animals given either buprenorphine, ketorolac or dexmedetomidine were compared to controls injected with sterile seawater with independent-samples *t*-tests. Sample sizes in this experiment are small (4 squid per group), thus statistical comparisons should be interpreted with caution.

In the general anesthesia trials, behavioral measures were recorded as latencies from drug wash-in and wash-out, and were compared among age classes and between the different drugs, with independent samples-*t*-tests. We recorded latencies to color change on defined bodily areas as outward indicators of progressive anesthesia induction and reversal, and also recorded the latency from wash-in of anesthetic bath solutions until no response was found to visual and gentle mechanical stimulation, which we used as our indicator of “full anesthesia”. We recorded the latency from washout until “full recovery” as the point at which the animal showed normal, coordinated swimming (synchronized fin beats, normal orientation without rocking or spinning, no slack tentacles or arms, and no abberant adhesion to surfaces), and rapid and normal responses to visual stimulation. Note that these measures typically lagged behind the body pattern indicators (arm color, mantle color, head-bar color).

All statistical analyses were conducted in Prism 9.4 (GraphPad, USA). The critical alpha for all comparisons was set at 0.05, and all *p*-values reported are two-tailed and corrected for multiple comparisons where appropriate.

## 3. Results

### 3.1. Nociceptive Threshold Testing

**Figure 2:**
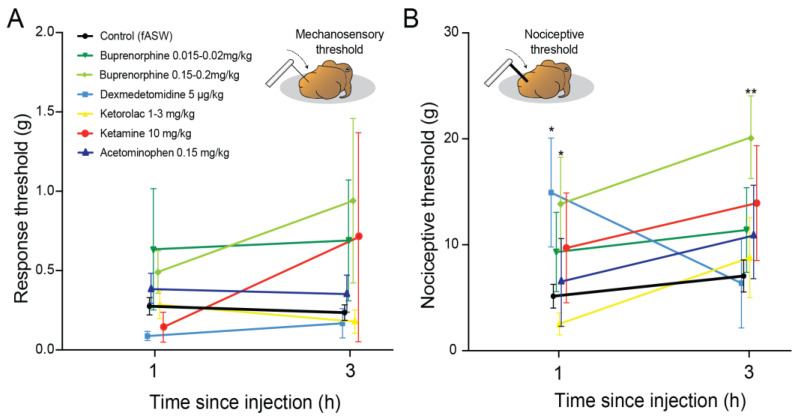
Responses of uninjured, healthy squid to applications of an ascending series of von Frey filaments after receiving candidate analgesics. (**A**) Response thresholds (color change, movement of individual body parts, or slow avoidance movements) were tested at one and three hours after drug dosing. Responses were typically variable and no significant effects were found compared with control squid injected with sterile seawater (unpaired *t*-tests, all NS). Refer to Table 2 for sample sizes. (**B**) Nociceptive thresholds were determined by recording instances of either inking, reflexive, high-speed escape jetting, or stimulus-directed arm grooming. At one hour nociceptive thresholds were elevated in squid given dexmedetomidine (*p* = 0.0071) and high-dose buprenorphine (*p* = 0.0098), compared with control squid (unpaired, Bonferroni-corrected *t*-tests). At three hours only squid given high dose buprenorphine showed continued elevation of nociceptive thresholds (*p* = 0.0071). Points show mean and error bars show standard error of the mean (SEM). * *p* < 0.05. ** *p* < 0.01).

For detection thresholds, one-way ANOVA of responses at 1 h indicated no overall significance (F_7,95_ = 1.17, *p* = 0.35, Figure 2A), which was similar at 3 h (F_7,94_ = 1.18, *p* = 0.31, Figure 2A). One-way ANOVA of nociceptive thresholds at one hour showed significant differences (F_7,93_ = 2.69, *p* = 0.013, Figure 2B). Post-hoc, pairwise comparisons revealed significantly higher thresholds for Dexmedetomidine-dosed squid compared with controls (*p* = 0.0071), and for squid dosed with buprenorphine at 0.15 mg/kg (*p* = 0.0098). At three hours, the one-way ANOVA remained significant (F_7,94_ = 2.44, *p* = 0.024), and pairwise comparisons showed the source of this effect was significantly higher thresholds in squid dosed with 0.15 mg/kg buprenorphine (*p* = 0.0071). We chose to advance buprenorphine and dexmedetomidine to the electrophysiological stage of the study, along with ketorolac. Although ketorolac did not show significant changes to nociceptive thresholds we observed some squid showing signs of reduced responsiveness which we suspected might equate to behavioral analgesia. No other drugs showed outward signs of efficacy.

Data for total trial were not normally distributed, thus we used non-parametric statistics to assess whether there was evidence for general sedative or hypnotic effects of the drugs we tested. We found no evidence of differences in total trial length among the drugs tested (Kruskall-Wallis test, *p* = 0.29).

### 3.2. Peripheral Nerve Excitability

**Figure 3:**
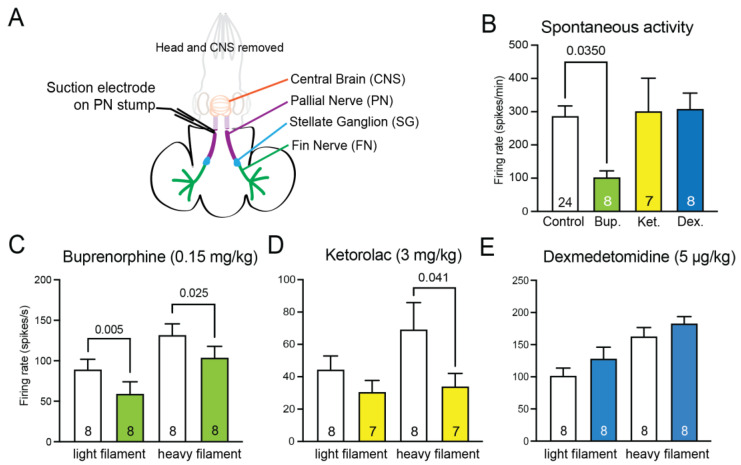
Peripheral nervous system excitability is suppressed by systemic analgesics. (**A**) A schematic of the nerve/tissue prep. The mantle was split down the midline and left and right sides were paired drug/control replicates. Drug-treated preparations were incubated in the same drug concentrations as shown in Figure 2, and controls were incubated in filtered artificial seawater. The large pallial nerve carries information to the central brain from the mantle nerves, which converge into the stellate ganglion. A suction electrode on the pallial nerve measures activity in response to stimulation on the mantle and fin tissue with von Frey filaments. (**B**) Spontaneous afferent firing was recorded for one minute prior to any stimulation being delivered. Only high-dose buprenorphine suppressed spontaneous activity (unpaired *t*-test vs control). (**C**) Buprenorphine suppressed evoked firing in response to touch with a light von Frey filament, which activates mechanoreceptors, and with a stiff von Frey filament, which activates nociceptors (paired *t*-tests vs. control-side prep). (**D**) Ketorolac suppressed nociceptor firing but had no effect on low-threshold mechanoreceptors. (**E**) Dexmedetomidine had no effect on pallial nerve activity. Bars show mean and error bars show standard error of the mean (SEM).

Recordings from the pallial nerve in isolated tissue-nerve preparations (Figure 3A) showed varied effects for the different drugs. Recordings of spontaneous afferent firing show significantly reduced firing frequency in the presence of buprenorphine vs. seawater controls (*p* = 0.035, unpaired *t*-test, Figure 3B), but no effect of either dexmedetomidine or ketorolac. Responses to touch on the mantle surface by either a light or heavy von Frey filament (activating low-threshold mechanoreceptors and nociceptors, respectively), showed a significant depressing effect of buprenorphine on firing for both light and heavy touch (Figure 3C, paired *t* test, *p* = 0.005 for light, *p* = 0.025 for heavy). Ketorolac also has a depressing effect on response to the heavy filament application (*p* = 0.041, paired *t* test, Figure 3D), but there was no effect of pallial nerve excitability after incubation with dexmedetomidine (Figure 3E).

### 3.3. Behavioral Response to Painful Sensory Input

**Figure 4:**
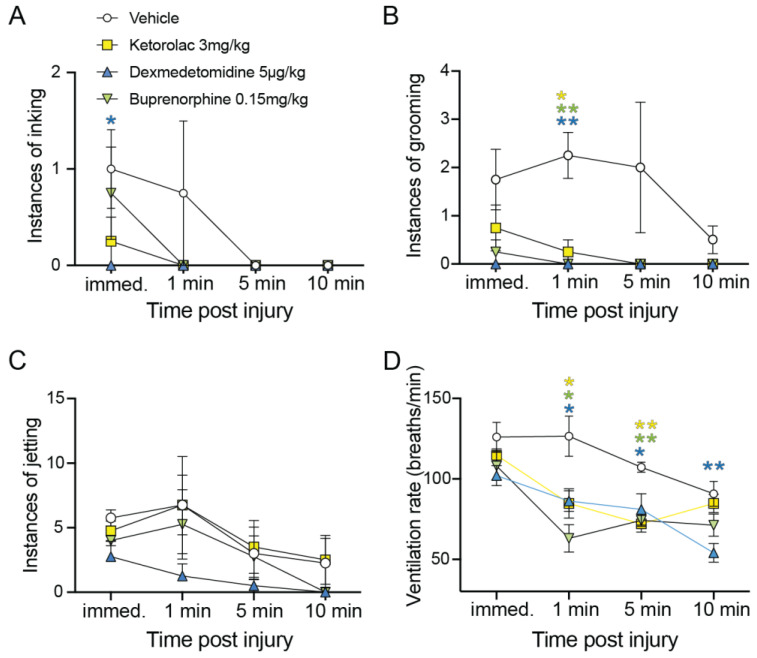
Pain-like behavior is suppressed by systemic analgesics. (**A**) Number of individual ink plumes released in response to fin pinch, and at one, five and ten minute intervals thereafter. Only dexmedetomidine significantly reduced inking (*p* = 0.04, unpaired *t*-test), although overall the instances of inking were low and these data show likely floor effects. (**B**) Site-directed grooming with the arms occurred repeatedly in control squid, but was significantly suppressed by all three drugs at one minute post-injury (Ket, *p* = 0.01, Dex, *p* = 0.003, Bup, *p* = 0.003, Bonferroni-corrected, unpaired *t*-tests). (**C**) Jetting showed no significant effects at any time point. (**D**) Ventilation rate, which is used as an indicator of pain in fish, was significantly higher in control squid at one and five minutes post-pinch compared with all three drug-treated groups (control vs. ketorolac, *p* = 0.03. Control vs. dexmedetomidine, *p* = 0.02, control vs. buprenorphine, *p* = 0.005, (**D**)). The suppression of respiratory rate was still present at five minutes for all drugs (control vs. ketorolac, *p* = 0.001. Control vs. dexmedetomidine, *p* = 0.04, control vs. buprenorphine, 0.004), but only for dexmedetomidine at 10 min post-pinch (vs. control, *p* = 0.008, (**D**)). Points show mean and error bars are standard error of the mean (SEM). * *p*, 0.05. ** *p*, 0.01. Colors of asterisks show comparisons of each color matched dataset to control.

Immediate inking in response to pinch was significantly less likely for squid injected with dexmedetomidine (*p* = 0.04, unpaired *t*-test, Figure 4A), but not for the other two drugs tested. Significant reduction in grooming behavior was found at one minute post-injury for ketorolac (*p* = 0.01, unpaired *t*-test), buprenorphine (*p* = 0.003) and dexmedetomidine (*p* = 0.003, unpaired *t*-tests, Figure 4B). There were no differences overall in instances of jetting among the groups (4C). A significant effect on ventilation rate for all three drugs was present at one minute (control vs. ketorolac, *p* = 0.03. Control vs. dexmedetomidine, *p* = 0.02, control vs. buprenorphine, *p* = 0.005, Figure 4D). The suppression of respiratory rate was still present at five minutes for all drugs (control vs. ketorolac, *p* = 0.001. Control vs. dexmedetomidine, *p* = 0.04, control vs. buprenorphine, 0.004), but only for dexmedetomidine at 10 min post-pinch (vs. control, *p* = 0.008, Figure 4D).

### 3.4. Sequence Alignments

**Figure 5:**
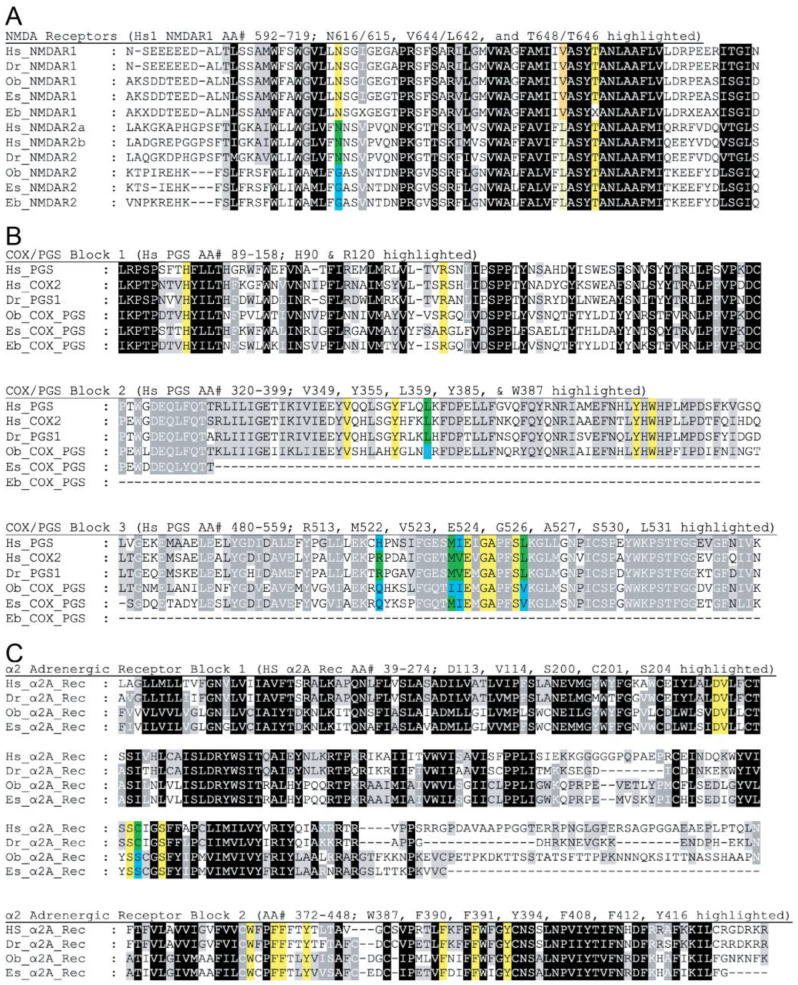
Multiple sequence alignments of putative receptors for Ketamine (**A**), Ketorolac (**B**), and Dexmedetomidine (**C**). Residues with described functions in vertebrates are highlighted in yellow if they are conserved in cephalopod sequences or highlighted in blue and green if they diverge. (**A**) Homologs for NMDAR receptors 1 and 2 are conserved across mammals and cephalopod species. Some residues surrounding the central vestibule containing the ketamine binding pocket [41] of NMDAR 1 (Hs_NMDAR1 T648-dark yellow, V644-dark yellow) and NMDAR 2 (Hs_NMDAR2 T646-bright yellow, L642-pale yellow) are conserved across species for which we have sequence data (Eb_NMDAR1 sequence quality was too low to confirm conservation of T648). NMDAR 1 exhibits conservation of N616 (bright yellow), but the conservation of the homologous site (N615) in NMDAR 2 was limited to vertebrates (*H. sapiens* and *D. rerio*), with a cephalopod glycine (bright blue) replacing the vertebrate asparagine (bright green). (**B**) The amino acid sequences for cephalopod cyclooxygenase-2/prostaglandin synthase (COX/PGS) are conserved with those of vertebrates (*H. sapiens* and *D. rerio*). Amino acid residues that line the cyclooxygenase channel in *H. sapiens* prostaglandin synthase (H90, R120, Y355, Y387, R513, E524, S530, and L531) or make contact with the NSAID Ibuprofen [42] (V359, L359, M522, V523, G526, and A527) were assessed for conservation. Complete conservation across cephalopods for which sequences were available) and vertebrates was observed in ten of these fifteen residues (bright yellow), while L359, R513, M522, V523, and L531 were not conserved in all cephalopods (bright blue and green residues). (**C**) The amino acid sequences for cephalopod (Ob and Es) alpha 2 Adrenergic (α2A) receptors are highly conserved with those of vertebrates (Hs and Dr). Amino acid residues known to line the binding pocket and play some role in ligand or activity [43] were assessed for conservation. Complete conservation across cephalopods (for which sequences were available) and vertebrates was observed in eleven of the twelve residues (yellow), and one residue (C201) was replaced by a Serine in cephalopods. Abbreviations: Cephalopods—Ob: *Octopus bimaculoides*; Es: *Euprymna scolopes*; and Eb: *Euprymna berryi*. Vertebrates—Hs: *Homo sapiens*; Dr: *Danio rerio*. NMDAR: N-methyl-D-aspartate receptor, PGS: prostaglandin synthase, COX: Cyclooxygenase, α2A_Rec: Alpha 2 Adrenergic Receptor.

Multiple sequence alignments revealed conserved cephalopod homologs for known or hypothesized vertebrate receptors for Ketamine (NMDAR [41] Ketorolac (cyclooxygenase2/prostaglandin synthase (COX2/PGS, [42] and Dexmedetomidine (α2A receptor) [43] Figure 5), suggesting potential mechanisms for each drug. We were not able to conclusively identify cephalopod homologs of the canonical target for Buprenorphine, the μ-opioid receptor.

### 3.5. General Anesthesia

**Figure 6:**
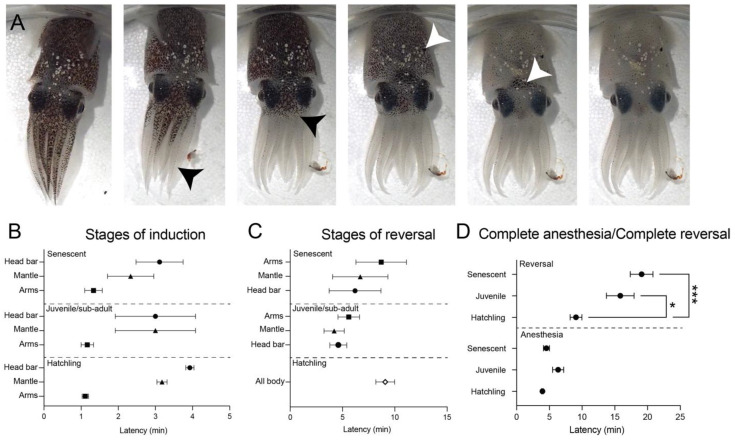
General anesthesia in multiple age-classes of *E. berryi* with a combination of 1% EtoH (*v*/*v*) and 1:3 ratio of isotonic MgCl2:SW. (**A**) Photographic sequence showing progressive outward signs of anesthesia induction. In all age classes paling of the arms progressed from tip to base (Black arrowheads), which was followed by all-over mantle paling (white arrowhead, 4th panel) and finally relaxation of chromatophores across the head-bar, between the eyes (5th panel). Once squid were completely pale, we tested for complete anesthesia by recording absence of response to visual stimulation, vibratory stimulation, or light touch on the body surface. Latency between complete paling and full anesthesia was variable. (**B**) Comparisons among the three age classes tested showed no significant differences in latency to paling of arms, mantle or head bars. (**C**) Reversal times were counted from the point where solutions were changed to fresh seawater. Return of chromatophore tone followed the reverse order of induction. In hatchlings we were only able to reliably identify whole-body darkening on recovery. No significant differences between age classes were found. (**D**) Latency to complete anesthesia and complete reversal wee compared among the age classes. No differences in induction times were found, but hatchlings recovered significantly faster than juvenile and senescent squid (one-way ANOVA followed by post-hoc Bonferroni corrected *t*-tests, critical alpha *p* < 0.05). Points show mean and error bars are standard error of the mean (SEM). * *p* < 0.05. *** *p* < 0.01).

Squid in all three age classes were effectively anesthetized with a combination of 1:3 MgCl_2_:ASW combined with 1% EtOH by volume. Behavioral signs of anesthesia were similar for all age classes, characterized by progressive paling of chromatophores starting at the tips of the arms and ending with relaxation of the dark head-bar (Figure 6A). This pattern occurred in reverse order during anesthesia reversal. All squid recovered after full anesthesia except for one hatchling, which died during anesthesia induction, and all squid who underwent successful reversal of anesthesia were alive 24 *h* later. Induction indicators (Figure 6B) and reversal indicators (Figure 6C) did not vary significantly among the different age classes, but time to full recovery was significantly longer for juvenile and senescent squid compared with hatchlings (one-way ANOVA, F(2,29) = 15.28, *p* < 0.0001, post-hoc *t*-tests with Bonferroni correction: Hatchling vs. juvenile, *p* = 0.01. Hatchling vs. senescent adults, *p* < 0.0001).

## 4. Discussion

Here we show promising evidence that drugs from several different drug classes may act as long-lasting, systemic analgesics for cephalopods undergoing potentially painful and distressing procedures. To our knowledge this is the first study to demonstrate welfare-related effects of analgesic drugs in any cephalopod. We also show that a previously-validated protocol for general anesthesia is effective in sepiolid squid of different age classes.

Recent studies aimed at identifying sentience, emotional capacity and affective state in invertebrates suggest that invertebrates may experience PSDLH [31] as a result of invasive procedures. However, there has been very minimal study of analgesia in any invertebrate species to date, which makes effective management of welfare challenging. We tested analgesic efficacy in multiple ways; first by measuring elevation of baseline (uninjured) nociceptive thresholds. This behavioral procedure produced highly variable outcomes that were not necessarily supported by electrophysiology data. The lack of effect in these initial screening tests may have led us to eliminate drugs with genuine analgesic properties, but we chose this conservative approach with the explicit goal of limiting potential suffering in this study. We also limited the range of different dosages we tested for each drug, beginning with a dose in the mid- to high-range reported as effective in fish (if available) or rodents. Given that very little is known about possible analgesic receptor distribution and affinity in cephalopods, it is also very plausible that the doses we tested were too low in some cases to identify significant effects. Thus we do not necessarily discount the possibility that the unsuccessful drugs we tested in the first phase of the study may be effective at different doses.

Among the analgesic candidates we tested we found evidence for elevated nociceptive threshold, behavioral analgesia and suppression of peripheral nerve excitability for buprenorphine only. Ketorolac, a potent NSAID, produced behavioral analgesia and electrophysiological evidence of peripheral neural effects, but did not significantly change baseline nociceptive thresholds. Dexmedetomidine produced elevated nociceptive threshold and behavioral analgesia, but no evidence of reduced excitability in the PNS. We suggest, therefore, that opioids and NSAIDs may have both peripheral and central models of action, whereas the a2 adrenoreceptor antagonist may act primarily centrally. The molecular targets of all three drugs in cephalopods are currently not known. Our bioinformatic analysis provided mixed support for the behavioral observations. We found no clear homolog of an opioid receptor in cephalopods, suggesting that buprenorphine exerts its effects through a different target, perhaps binding to targets of met-enkephalin or somatostatins. In vertebrates, buprenorphine binds to mu, delta and kappa opioid receptors, and the less well studied nociceptin (ORL-1/NOP) receptor [44,45,46], but these did not produce strong alignments with cephalopod sequences. In general, there is a dearth of support for the existence of any opioid receptors in cephalopods.

Ketorolac is a veterinary NSAID with potent analgesic and anti-inflammatory properties. It is a non-selective COX inhibitor that is often used in combination with opioids and other analgesics [47,48] to control post-operative pain in companion animals. To our knowledge there are no previous studies of its analgesic effects in invertebrate animals. In this study we found strong evidence for a suppressing effect on nociceptor excitability which may be mediated via the same molecular target as in mammals, given that our bioinformatic analysis shows good alignment with vertebrate COX (prostaglandin endoperoxide synthase) and cephalopod prostaglandin g/h synthase, including at the putative binding sites. Unlike for dexmedetmodine and buprenorphine, we found no evidence for changes in baseline nociceptive thresholds, which also means that we do not have an indication of ketorolac’s temporal properties in cephalopods, as this stage of the study also provided repeated, time-based measures. Additional study of dosing and duration of effect are needed.

Dexmedetomidine is an agonist of the alpha2 adrenergic receptor in vertebrates, where its binding causes membrane hyperpolarization and inhibition of release of glutamate, an excitatory neurotransmitter [49]. In a pilot study in the snail *Lymnaea stagnalis*, dexmedetomidine blocked excitatory cholinergic neurotransmission, but the receptor to which the drug was presumably bound to produce this suppression remains unknown [50]. Bioinformatic analyses show that there is similarity between the vertebrate a2a receptor, putative a2a receptors in cephalopods, and octopamine and tyramine receptors in invertebrates. Drugs targeting octopamine receptors in invertebrate parasites have been shown to interact with the a2a receptor in mammals [51]. Taken together, these limited lines of evidence suggest dexmedetomidine may produce analgesia in cephalopods via interaction with octopaminergic neurotransmission, but this is speculative. Dexmedetomidine also had a quite short period of efficacy in tests of baseline nociceptive thresholds at one and three hours, suggesting that it may be displaced from its receptor target quickly. Rapid and short-acting analgesia is considered an advantageous aspect of dexmedetomidine in clinical practice, and that seemed to be similar in our study. Another widely recognized advantage of dexmedetomidine in clinical and veterinary use is the absence of respiratory depression common to other drugs. In contrast, we found that dexmedetomidine had the strongest effect on ventilation in our study, but we note also that this effect was in contrast to the elevated respiratory rate we considered evidence of pain, not compared with normal respiration.

Although our results supported sequence homology for receptors that may bind three of the drugs described in this study, each putative cephalopod receptor diverged from its vertebrate homolog at one or more of the previously described sites associated with ligand binding. It is also possible that even conserved residues may function differently in divergent structures of the cephalopod receptor homologs, meaning the mere existence of a cephalopod receptor homolog is far from sufficient to conclusively infer drug interactions. For example, conservation of some residues like the α2A Receptor residue Y394 may play no role in the function of partial agonists like Dexmedetomidine because this residue binds only to full agonists.

We also show that sepiolid squid can be safely and effectively anesthetized using a combination of 1% ethanol by volume mixed into a 1:3 mixture of MgCl2 and seawater. This combination produced reliable loss of responsiveness within 5 min in all age classes, and was very well tolerated, with only a single mortality in this part of the study. All the age classes showed consistent outward indications of anaesthesia progression, with progressive paling of the arms from tip to base, followed by paling of the mantle, and lastly the loss of the dark head-bar across the top of the head. Loss of the head bar was followed closely by loss of righting reflex and loss of behavioral response to light touch on the mantle. Among sub-adult and senescent squid, times to anesthesia and recovery were longer and more variable than those of hatchlings, but unlike in the cuttlefish, *Sepia officinalis* (Abbo et al., 2021), temporal patterns of anesthesia in senescent squid did not differ significantly from younger age-class animals. In octopuses, senescence is associated with a range of degenerative physiological and behavioral changes [52], which may correlate with differences in temporal patterns of anesthesia. The senescent squid in this study were in the early stages of senescence, indicated by less frequent burying behavior, less successful prey capture and onset of breeding behavior, but otherwise were in good physical condition. It is possible that later-stage senescent squid, which we did not use here, might show different responses to anesthesia, similar to previous findings in *S. offficinalis.*

The mode of general anesthesia action for both ethanol and MgCl_2_ in cephalopods remains somewhat unclear. In other species, ethanol binds to the GABA receptor, enhancing inhibitory transmission in a wide range of neural circuits, and this is likely similar in cephalopods. Ethanol also potentiates glycine currents and increases conductance of several types of potassium channels, all of which contribute to neuron hyperpolarization in mammals [53,54,55,56,57]. MgCl_2_ has multiple effects; acting generally to suppress sodium current and potentially facilitating the blockade of NMDA receptors by providing high concentrations o of magnesium ions, however, its precise mode and site of action in anesthesia in cephalopods is not firmly established [58]. In other studies where direct recording from the nervous system of cephalopods was conducted during anesthesia, both MgCl_2_ and ethanol were effective at blocking afferent and efferent neurotransmission from the brain and periphery. The neuroanatomy of sepiolid squid is less amenable to this type of minimally-invasive recoding, thus we did not attempt to record neural activity in this study. However, it is clear that this combination of drugs is effective for immobilization and sedation, and based on previous evidence from other cephalopod clades, we suggest that it is likely also effective for producing loss of consciousness and loss of sensation.

## 5. Conclusions

In this study we show that welfare enhancing interventions for sepiolid squid are readily achievable and are likely to be applicable to other cephalopod clades. We demonstrate that general anesthesia using a mix of MgCl_2_ and ethanol produced reliable and safe immobilization and sedation and likely also loss of sensation. Different age classes have somewhat different temporal patterns of anesthesia, although anesthesia in all age classes was successful. Importantly, for the first time we show evidence for systemic analgesia in cephalopods achieved with three distinct classes of drugs; an opioid, an NSAID and an alpha2 receptor antagonist, and suggest possible molecular targets through comparative bioinformatic analysis. Further study is needed to refine analgesic dosing and to test whether combinations of these or other drugs may provide longer or more effective analgesia.

We anticipate that this study should be of considerable value for cephalopod researchers performing invasive and potentially painful procedures, and for those working in nations where cephalopods are regulated by research animal welfare laws.

## Figures and Tables

**Figure 1:**
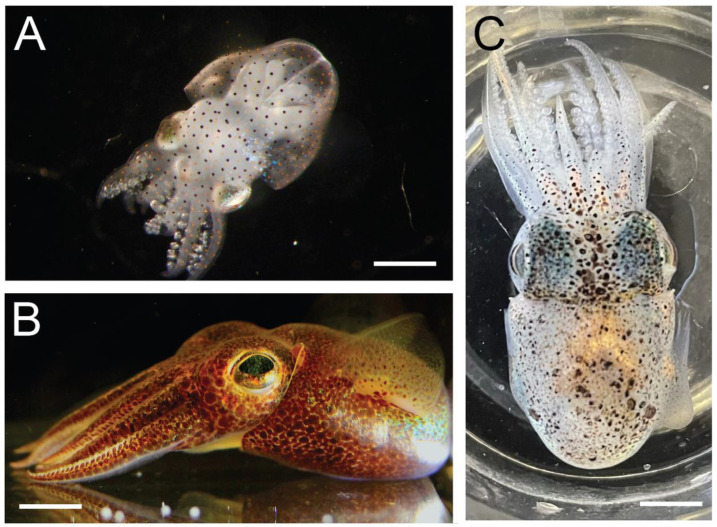
*Euprymna berryi*, (**A**) Hatchling (**B**) Pre-reproductive adult (**C**) Senescent adult. All squid were captive bred in the laboratory for this study. Scale bars are 500 µm (**A**), and 5 mm (**B**,**C**).

**Table 1:** Numbers of squid used in each phase of the study. Severity classification from [31].

Experiment Phase	Treatments	Count	Severity Classification
Analgesia: Nociceptive threshold testing	Control	51	Mild/Moderate
	Dexdomitor	6	
	Ketorolac	8	
	Buprenex (low dose)	10	
	Buprenex (high dose)	7	
	Acetominophen	6	
	Ketamine	6	
			*n* = 94
Analgesia: Electrophysiology	Ketorolac	8	Non-recovery
	Buprenex	8	
	Dexdomitor	8	
			*n* = 24
Analgesia: Pain behavior	Control	4	Moderate/Severe
	Buprenex	4	
	Dexdomitor	4	
	Ketorolac	4	
			*n* = 16
General anesthesia	Hatchling	16	Mild
	Juvenile/sub-adult	6	
	Senescent adult	11	
			*n* = 33
Total in Mild/Moderate			127
Total in Moderate/Severe			16
Total Non-recovery			24
Total squid used in this study			*n* = 167

**Table 2:** Candidate analgesic drugs tested for efficacy in *E. berryi*, their dosages and their administration route.

Agent	Schedule	Classification	Route	Dose	Predicted Target
Acetaminophen	Over the Counter (OTC)	Pain Reliever & Antipyretic	Oral (PO)	0.14 mg/kg	COX-2 prostaglandin pathway, other targets
Ketorolac	Prescription (Rx)	Non-Steroidal Anti-Inflammatory (NSAID)	Intramuscular (IM)	3–6 mg/kg	COX-2 prostaglandin pathway
Dexmedetomidine	Prescription (Rx)	Sedative	Intramuscular (IM)	0.005 mg/kg	Alpha-2 adrenoreceptor
Ketamine	Schedule Three (III)	Dissociative Anesthetic	Intramuscular (IM)	10 mg/kg	NMDA receptor, likely other targets
Buprenorphine	Schedule Three (III)	Opioid	Intramuscular (IM)	Low: 0.015 mg/kg	Mu-opioid and kappa-opioid receptors, possible met-enkephalin receptors
				High: 0.15 mg/kg	

## Data Availability

Raw data is available upon reasonable request from the corresponding author.
